# Low-frequency oscillations of finger skin blood flow during the initial stage of cold-induced vasodilation at different air temperatures

**DOI:** 10.1186/s40101-020-00248-4

**Published:** 2020-11-23

**Authors:** Toshihiro Sera, Taiki Kohno, Yusuke Nakashima, Musashi Uesugi, Susumu Kudo

**Affiliations:** 1 Department of Mechanical Engineering, Faculty of Engineering, Kyushu University, 744 Motooka, Nishi-ku, Fukuoka, 819-0395 Japan; 2 Department of Mechanical Engineering, Graduate School of Engineering, Kyushu University, Fukuoka, Japan; 3 Graduate School of Systems Life Science, Kyushu University, Fukuoka, Japan

**Keywords:** Cold-induced vasodilation, Finger skin blood flow, Wavelet analysis, Air temperature, Arteriovenous anastomoses

## Abstract

**Background:**

Cold-induced vasodilation (CIVD) is known to be influenced by the ambient temperature. Frequency analysis of blood flow provides information on physiological regulation of the cardiovascular system, such as myogenic, neurogenic, endothelial nitric oxide (NO) dependent, and NO-independent activities. In this study, we hypothesized that the major origin of CIVD occurs prior to the CIVD event and investigated finger skin blood flow during the initial stage of CIVD at different ambient temperatures using frequency analysis.

**Methods:**

Eighteen healthy volunteers immersed their fingers in 5 °C water at air temperatures of 20 °C and 25 °C. Finger skin blood flow was measured using laser Doppler flowmetry and analyzed using Morlet mother wavelet. We defined the time when the rate of blood flow increased dramatically as the onset of CIVD, and defined three phases as the periods from the onset of cooling to minimum blood flow (vasoconstriction), from minimum blood flow to the onset of CIVD (prior to CIVD), and from the onset of CIVD to maximum blood flow (CIVD).

**Results:**

The increment ratio of blood flow at CIVD was significantly higher at 20 °C air temperature. In particular, at 20 °C air temperature, arteriovenous anastomoses (AVAs) might be closed at baseline, as finger skin temperature was much lower than at 25 °C air temperature, and endothelial NO-independent activity was significantly higher and neurogenic activity significantly lower during vasoconstriction than at baseline. Additionally, the differences in both activities between vasoconstriction and prior to CIVD were significant. On the other hand, there were no significant differences in endothelial NO-dependent activity between baseline and all phases at both air temperatures.

**Conclusions:**

Our results indicated that the increase of endothelial NO-independent activity and the decrease of neurogenic activity may contribute to the high increment ratio of blood flow at CIVD at 20 °C air temperature.

## Background

In order to maintain thermal homeostasis, heat loss is reduced during direct local cooling by vasoconstriction of skin vessels, resulting in a decrease in finger skin blood flow. However, after several minutes of vasoconstriction, finger skin blood flow increases and decreases cyclically despite continuous local cooling. This behavior of the blood flow during local cooling is known as cold-induced vasodilation (CIVD) and was first reported as the “hunting response” [1]. CIVD may be considered a protective response to minimize the risk of cold injury in distal peripheral tissues [2, 3]. Arteriovenous anastomoses (AVAs) may be implicated in CIVD because CIVD mainly occurs where AVAs are abundant [4], and relaxation of AVAs leads to an increase in local blood flow and tissue temperature [5]. Differences in CIVD responses between people of different ages [6–8], people of different races [9, 10], and different O_2_ concentrations [11] and the effects of tobacco [12] and menthol activation [13] on CIVD responses have been investigated. In addition, some reviews of several potential mechanisms and models have been published [5, 14–16].

Some studies [17, 18] reported that ambient temperature affects CIVD and that a lower core temperature reduces the magnitude and frequency of CIVD [17]. In addition, the minimum and maximum temperatures during immersion of the fingers into cold water are higher when the core temperature is higher. Further, the onset time of CIVD, defined as the time taken for the initial increase in temperature after immersion, is much longer when the core and finger skin temperatures are low [17]. The influence of body thermal status on the CIVD response was investigated during mild hypothermia, thermoneutrality, and hyperthermia. The researchers reported that the onset of CIVD was significantly prolonged in hypothermia compared with thermoneutrality [18]. However, these studies only focused on changes in finger skin temperature during local cooling and not on skin blood flow.

Several methods are available to quantify the magnitude of CIVD in finger skin, and temperature is the most commonly used measurement. However, skin temperature is an indirect measurement of skin blood flow, with a time lag, although it can provide an average for a larger area. In addition, the measured skin temperature is a mixture of the temperatures of the skin and the surrounding medium, and careful attachment of the small thermal sensor is required. On the other hand, blood flow fluctuates in an oscillatory manner even at rest. The frequency of skin blood flow, particularly when measured directly by a laser Doppler velocimeter, provides information on the physiological regulation of the cardiovascular system; for example, 0.1 Hz indicates myogenic activity; 0.04 Hz, neurogenic activity; and 0.01 Hz, endothelial activity [19, 20]. In addition, low-frequency endothelial activity can be divided into two domains representing nitric oxide (NO) dependence and independence [21, 22]. Therefore, wavelet analysis of the blood flow measured by a laser Doppler velocimeter is useful to investigate the mechanisms of CIVD [23, 24]. In particular, a previous study focused on neural and endothelial activities reported that neurogenic activity was reduced in synchrony with the blood flow during hand cooling and subsequent CIVD, suggesting that sympathetic withdrawal directly contributes to the onset of CIVD [24]. However, we believed that the main origin of CIVD occurs prior to CIVD response, and we focused on the initial stage of CIVD. Additionally, a previous study reported that the fluctuations in blood velocity in the cooled finger stopped when the water bath temperature dropped below ~ 21.5 °C, although the fluctuations were unaltered during local cooling at water temperature of 35-27 °C, suggesting that the AVAs presumably remained closed at water temperatures below 21.5 °C [25, 26]. The response of endothelial-dependent vasodilation (EDV) at a high density of AVAs (finger pulp) differed between air temperatures of 29 °C and 22 °C [27]. Furthermore, the CIVD response is influenced by thermal sensations [28]. At air temperature of 25 °C, the participants felt “neither cool nor warm, and thermally comfortable,” on the other hand, the body continued to cool and the participants felt “slightly uncomfortable and cool” [28]. These results indicate that the CIVD responses may differ for different temperatures within the range of normal air temperature. In this study, we investigated differences in myogenic, neural, and endothelial activities during the initial stage of CIVD at air temperatures of 20 °C and 25 °C. We assumed that the increase in blood flow at CIVD were greater at 20 °C than at 25 °C since closed AVAs opened, and hypothesized that the changes in each activity during the initial CIVD response were also greater at 20 °C. To achieve this objective, healthy participants immersed their fingers in 5 °C water at air temperatures of 20 °C and 25 °C, and we analyzed finger skin blood flow during the initial stage of CIVD using wavelet analysis.

## Materials

### Participants

Eighteen healthy nonsmoking volunteers (16 males and 2 females, 18–25 years old) were recruited for the experiments. They had not been diagnosed with any cardiovascular disease and were not taking any medications. All participants provided informed consent, and the local ethics committee at Kyushu University approved the study (H24-02, 685-00). The participants refrained from alcohol intake and heavy exercise for 24 h prior to the study and avoided caffeine-containing drinks on the day of the experiments. To compare CIVD responses at air temperatures of 20 °C and 25 °C, the participants wore the same clothing, consisting of T-shirts, shorts, socks, and slippers, at both temperatures during the experiments, as the previous study [28]. The face, forearms, hands, shins, and calves were left uncovered during the measurements. The estimated clo value for these clothes was 0.27 [29].

### Instruments and protocol

During the experiments, finger skin blood flow was measured using laser Doppler flowmetry (LDF; FLO-C1, OMEGAWAVE, Japan). The probe for the blood flow (ML type, OMEGAWAVE, Japan) and the thermistor for the temperature (TSD202F, BIOPAC Systems, Inc., USA) were attached to the ventral aspect of the distal phalanx of the middle finger using surgical tape. The diameter and the penetration depth of the LDF probes were 15 and 1.0 mm, respectively, and the diameter of the skin temperature sensor was 9.8 mm. To reduce the risk of water intrusion between the probes and the skin and the influence of the medium temperature on the LDF measurement, the probes were covered by a custom-made heat insulator. The thermistor was connected to an amplifier (SKT100C, BIOPAC Systems, Inc., USA), and the finger skin blood flow and temperature were recorded at 200 Hz using a data acquisition and analysis program (MP150, BIOPAC Systems, Inc., USA). LDF data were expressed in arbitrary units (AU).

The room temperature was set to 20 °C or 25 °C as appropriate. The order of experiments was random, and two trials were conducted on each subject on different days. The participants sat on a sofa with a backrest during the experiments. They rested for at least 30 min prior to data collection to confirm that all parameters were stable before beginning the experiments. Subsequently, the participants rested quietly for 10 min for the baseline data, then immersed their middle finger up to the second phalanx for 30 min in a custom-made cold-water bath at 5 °C designed to expose the finger to local cooling, without moving the finger. They then withdrew the finger and rested quietly again for 20 min. In addition to finger skin blood flow and temperature, sublingual temperature (Tempa•DOT™, Medical Indicators, Inc., USA) was measured before and after the experiments. The participants were instructed to place the sensor under their tongue as far back as possible and to close the mouth for 60 s without speaking.

Previously, a single digit, typically the index or middle finger, was usually immersed in cold water for CIVD research [14]. However, most recent protocols have used full hand immersion [16], as the CIVD response is highly variable across the fingers [30]. On the other hand, the difference in endothelial-dependent vasodilation (EDV) response between 29 and 22 °C was dependent on the density of AVAs, and at 22 °C the EDV response was higher at high density of AVAs (finger pulp), whereas the response was equal at low density of AVAs (wrist skin) [27]. In this study, we also investigated the difference in skin blood flow between air temperatures of 20 °C and 25 °C, and therefore, we used only the middle finger immersion for local cooling.

### Data analysis

The frequency bands and their associated physiological functions were 0.005–0.01 (endothelial NO-independent activity), 0.01–0.02 (endothelial NO-dependent activity), 0.02–0.06 (neurogenic activity), and 0.06–0.15 Hz (myogenic activity) [19, 22, 24, 31]. In this study, after downsampling to 5 Hz using the AcqKnowledge software (BIOPAC Systems, Inc., USA), a Morlet mother wavelet was used to perform wavelet analysis on the LDF signal using the Biomass program (ELMEC Inc., Japan) to investigate which component dominates CIVD. As previous work reported that a minimum of 20 min of data was required for low-frequency resolution [20], we used 60 min of data for wavelet analysis. The wavelet amplitudes were presented as a ratio of the total power between 0.005 and 0.15 Hz.

**Fig. 1:**
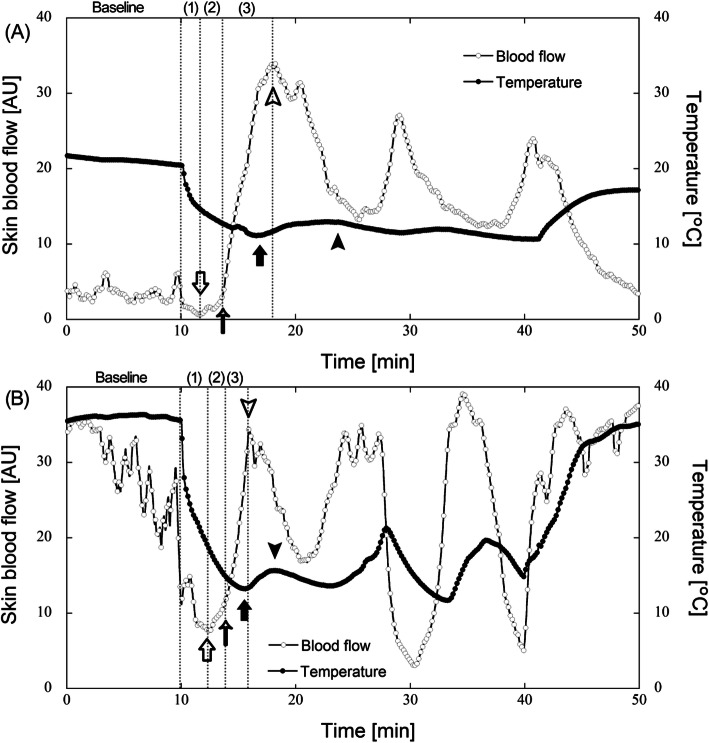
Representative blood flow and temperature in response to finger cooling at 20 °C (**a**) and 25 °C (**b**) air temperatures. The finger was exposed to local cooling at 5 °C for 30 min following 10 min of rest. The first maximum and minimum temperatures are indicated by black arrows and arrowheads, respectively, suggesting the presence of a CIVD event. The first maximum and minimum blood flows are indicated by white arrowheads and arrows, respectively. In addition, the onset time of the CIVD (CIVD onset_sbf) is indicated by small arrows. Phase 1 (vasoconstriction; from the onset of cooling to minimum blood flow), phase 2 (prior to CIVD; from minimum blood flow to CIVD onset_sbf), and phase 3 (CIVD; from CIVD onset_sbf to maximum blood flow) are indicated as (1–3), respectively.

Figure 1 shows representative finger skin blood flow and temperature in response to finger cooling at 20 °C (A) and 25 °C (B) air temperatures. A CIVD event was determined as a minimum increase in finger temperature after the blood flow reached the minimum first, due to local cooling, and then increased. We recorded the minimum and maximum blood flow and finger temperature associated with the CIVD event, and particularly the increment ratio in blood flow at CIVD was defined as the ratio of maximum to baseline blood flow. Previously, the onset time of CIVD was defined as the time from immersion to the minimum skin temperature (CIVD onset_sk) [11, 14, 17, 18]. This definition is reasonable, but the period from immersion to CIVD cannot be separated into only two phases by defining vasoconstriction as the phase from immersion to the minimum temperature and CIVD as the phase from the minimum to maximum temperatures. Alternatively, the wavelet amplitudes at maximum and minimum blood flows were evaluated, and minimum blood flow was considered at the event prior to CIVD [24]. However, we think this event prior to CIVD is dominated by vasoconstriction due to local cooling. On the other hand, in this study, we found that blood flow did not increase linearly from the minimum to the maximum flow but initially increased slowly and/or fluctuated and then began to increase rapidly (Fig. 1). We calculated the maximum and averaged gradients (*δ*_*max*_ and *δ*_*ave*_) between two adjacent data from minimum to maximum blood flow. The minimum temperature generally occurs before maximum blood flow [24] (Fig. 1), and therefore, to distinguish the phase prior to CIVD from vasoconstriction, we defined the time when the gradient was (*δ*_*max*_ + *δ*_*min*_)/2, and thereafter the gradient did not decrease until the minimum temperature, as the onset of the CIVD event (CIVD onset_sbf), and we defined phases 1, 2, and 3 as the periods from the onset of cooling to minimum blood flow (vasoconstriction), from minimum blood flow to CIVD onset_sbf (prior to CIVD), and from CIVD onset_sbf to maximum blood flow (CIVD), respectively (Fig. 1). At both air temperatures, the temperature decreased with local cooling and then increased, which indicated the presence of a CIVD event. Additionally, before the CIVD event, vasoconstriction and vasodilation occurred, indicated by white arrows and arrowheads (Fig. 1). The increase in the rate of blood flow was smaller soon after vasoconstriction than before vasodilation, and CIVD onset_sbf is indicated by small arrows (Fig. 1). We evaluated the average wavelet amplitude at baseline before cooling and before each phase, and the baseline and each phase are shown in Fig. 1.

Analysis of the significance of differences in specific events (level: baseline, minimum, and maximum) in skin temperature and blood flow between different air temperatures was performed using two-way repeated-measures analysis of variance (two-way RM ANOVA). Analysis of the significance of differences in phases based on blood flow (level: baseline, phases 1–3) between different air temperatures was also performed using two-way RM ANOVA. When significant differences were found, all combinations were analyzed by Tukey-Kramer multiple comparison post hoc tests. The increment ratio in blood flow during CIVD between air temperatures was evaluated using the paired *t* test. Statistical significance was set to *p* < 0.05.

## Results

Fourteen and 16 participants experienced at least one CIVD event at air temperatures of 20 °C and 25 °C, respectively. The average sublingual temperature was 36.5 °C ± 0.1 °C before and after the experiments at 20 °C air temperature. At 25 °C air temperature, the average sublingual temperature was 36.7 °C ± 0.1 °C and 36.6 °C ± 0.1 °C before and after the experiments, respectively. The sublingual temperatures were constant between the air temperatures and between the periods before and after the experiments.

**Fig. 2:**
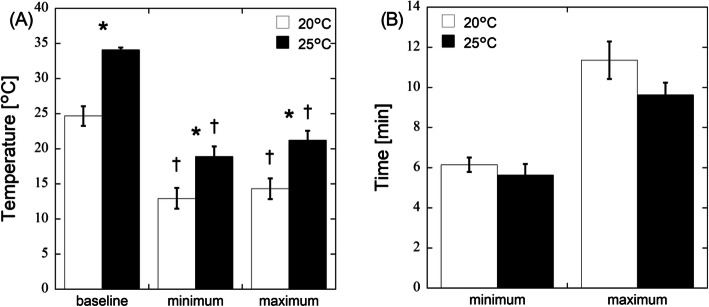
Finger skin temperature during baseline and CIVD at air temperatures of 20 °C and 25 °C. The minimum and maximum temperatures are shown as min and max, respectively (**a**). The time points from the onset of local cooling to the minimum and maximum temperatures are shown in (**b**). Data are presented as the average value ± SE. *p < 0.05 from 20 °C air temperature, and ^†^*p* < 0.05 from baseline at the same air temperature, respectively

**Fig. 3:**
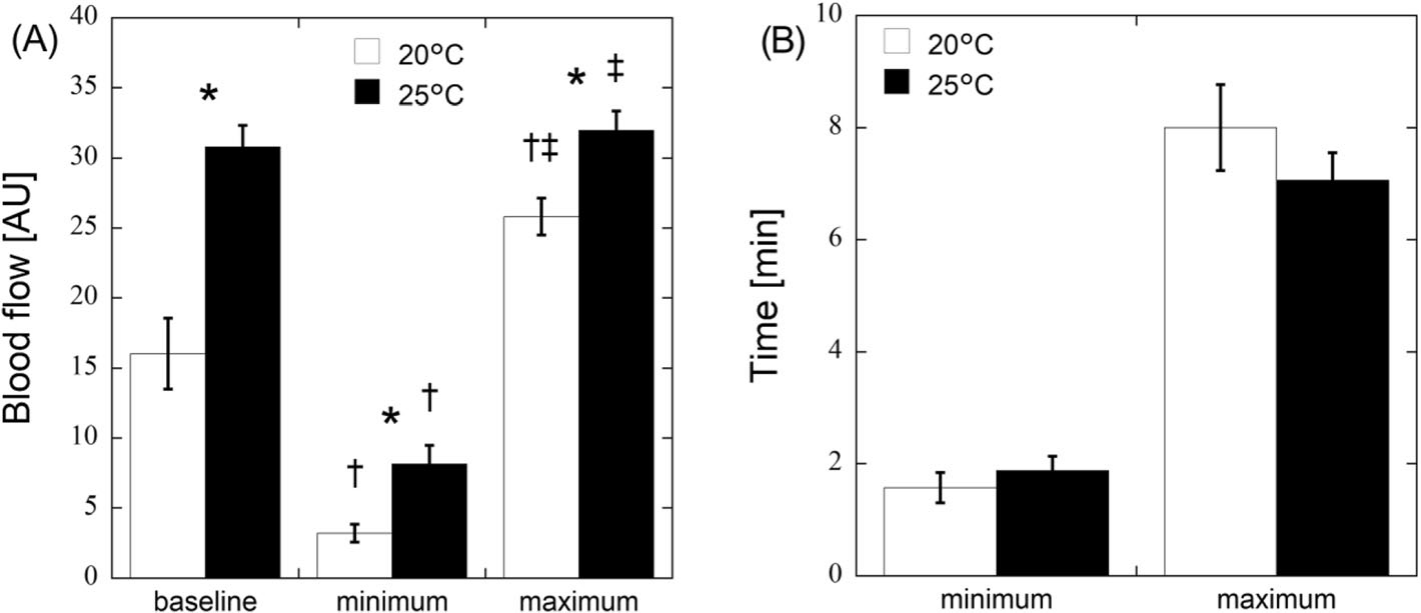
Finger skin blood flow during baseline and CIVD at air temperatures of 20 °C and 25 °C. The minimum and maximum blood flows are shown as min and max, respectively (**a**). The time points from the onset of local cooling at min and max are shown in (**b**). Data are presented as the average value ± SE. *p < 0.05 from 20 °C air temperature, ^†^*p* < 0.05 from baseline at same air temperature, and ^‡^*p* < 0.05 from minimum at the same air temperature, respectively

**Fig. 4:**
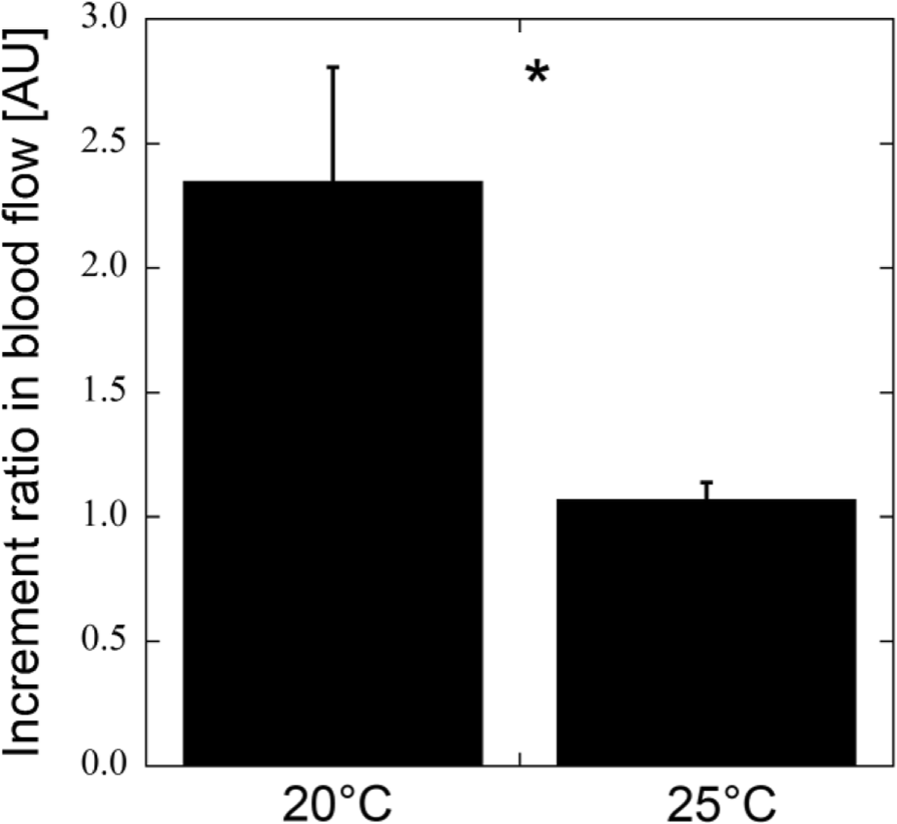
Increment ratio in blood flow at CIVD, defined as the ratio of the maximum blood flow to baseline. Data are presented as the average value ± SE. *p < 0.05 from 20 °C air temperature

Figure 2a shows the finger skin temperature at baseline and CIVD at air temperatures of 20 °C and 25 °C. The baseline, minimum, and maximum temperatures were 24.7 °C ± 1.4 °C, 12.9 °C ± 1.5 °C, and 14.3 °C ± 1.3 °C at 20 °C air temperature and 34.1 °C ± 0.3 °C, 18.9 °C ± 1.4 °C, and 21.2 °C ± 1.3 °C at 25 °C air temperature, respectively. The minimum and maximum temperatures were significantly lower than baseline at both air temperatures (*p* < 0.001). The finger skin temperatures were significantly higher at 25 °C air temperature than at 20 °C (*p* < 0.003). Figure 2b shows the time points for the CIVD event. The time was 6.1 ± 0.4 min and 11.4 ± 0.9 min from the onset of cooling to the minimum and maximum temperatures at 20 °C air temperature, respectively. At 25 °C air temperature, the time was 5.6 ± 0.6 min and 9.6 ± 0.6 min at the minimum and maximum temperatures. The CIVD event occurred slightly earlier at 25 °C air temperature. Figure 3a shows the baseline, minimum, and maximum blood flow at air temperatures of 20 °C and 25 °C. The baseline, minimum, and maximum blood flow was 16.0 ± 2.5, 3.2 ± 0.6, and 25.8 ± 1.3 AU at 20 °C air temperature and 30.8 ± 1.5, 8.1 ± 1.3, and 32.0 ± 1.4 AU at 25 °C air temperature, respectively. The minimum and maximum blood flows were significantly lower and higher than baseline at 20 °C air temperature, respectively (*p* < 0.001). The maximum blood flow was almost the same as the baseline blood flow at 25 °C air temperature. The blood flow at baseline was significantly lower at 20 °C air temperature (*p* < 0.001), resulting in a much lower finger skin temperature (Fig. 2a). The time was 1.6 ± 0.3 min and 8.0 ± 0.8 min from the onset of cooling to the minimum and maximum blood flows at 20 °C air temperature, respectively (Fig. 3b). At 25 °C air temperature, the time was 1.9 ± 0.3 min and 7.1 ± 0.5 min at the minimum and maximum temperatures (Fig. 3b). The skin blood flow reached the maximum slightly earlier at 25 °C air temperature. Figure 4 shows the increment ratio in blood flow at CIVD, and the blood flow during CIVD increased by 2.3 ± 0.5 times at 20 °C air temperature.

**Fig. 5:**
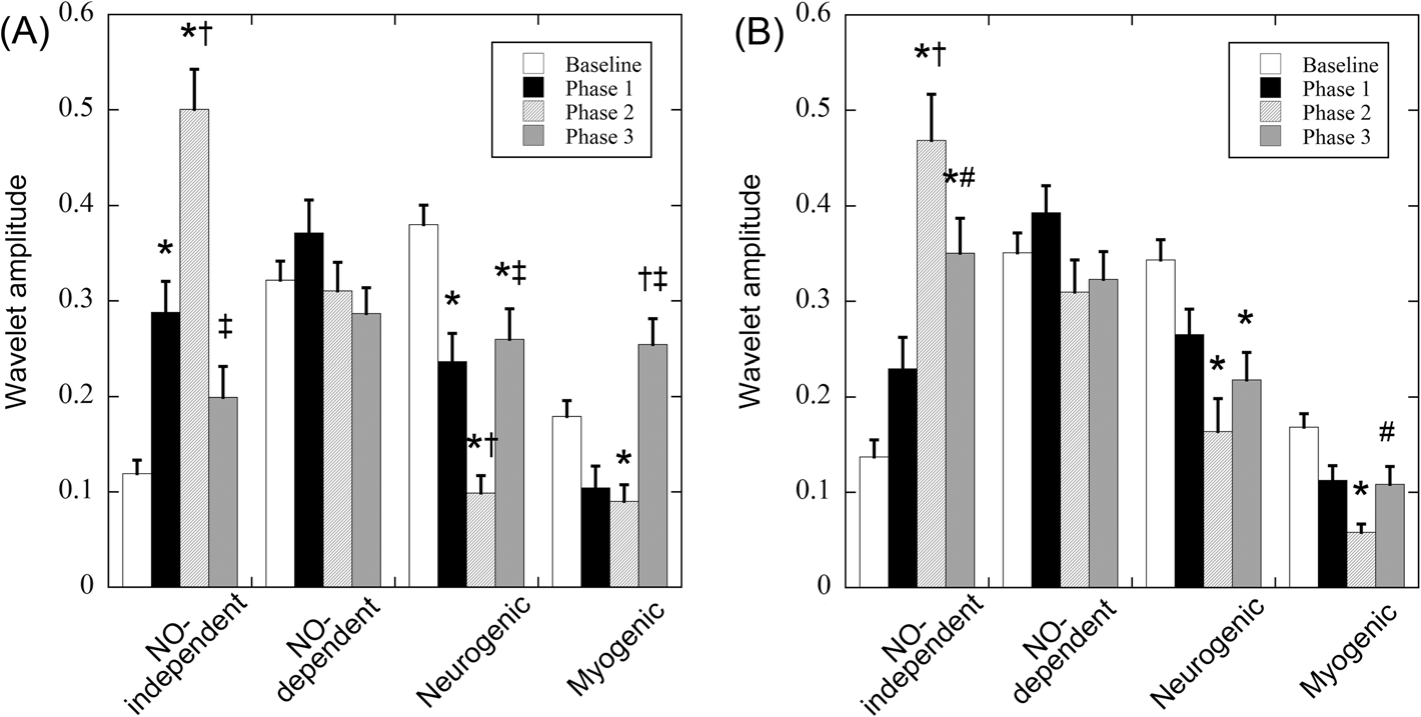
Changes in the wavelet amplitudes of endothelial NO independent, endothelial NO dependent, neurogenic, and myogenic activities at 20 °C (**a**) and 25 °C (**b**) air temperatures. Phases 1, 2, and 3 are at vasoconstriction, prior to CIVD, and at CIVD, respectively, shown in Fig. 1. Data are presented as average values ± SE. *p < 0.05 from baseline, ^†^*p* < 0.05 from phase 1, ^‡^*p* < 0.05 from phase 2, and ^#^*p* < 0.05 from the same phase at 20 °C air temperature, respectively

Figure 5 shows the changes in the average wavelet amplitudes of endothelial NO independent, endothelial NO dependent, neurogenic, and myogenic activities at baseline and each phase at 20 °C (A) and 25 °C (B) air temperatures. At 20 °C air temperature (Fig. 5a), the wavelet amplitude for endothelial NO-independent activity was 0.12 ± 0.01 at baseline and significantly increased at phase 1 compared with that at baseline (*p* = 0.004). The amplitude at phase 2 was 0.50 ± 0.04, which was significantly higher than that at baseline and phase 1 (*p* < 0.001). The amplitude at phase 3 decreased significantly compared with that at phase 2 (*p* < 0.001) but tended to be higher than that at baseline. The amplitude of endothelial NO-dependent activity was 0.32 ± 0.02 at baseline, and there were no significant differences in amplitude between baseline and any of the phases. The amplitude of neurogenic activity was 0.38 ± 0.02 at baseline and decreased significantly at phase 1 compared with that at baseline (*p* = 0.002). The amplitude at phase 2 was 0.10 ± 0.02, which was significantly smaller than the amplitudes at baseline and phase 1 (*p* < 0.003). On the contrary, the amplitude at phase 3 was significantly higher than that at phase 2 (*p* < 0.001) but was significantly smaller than that at baseline (*p* = 0.01). The amplitude of myogenic activity was 0.18 ± 0.02 at baseline and tended to decrease from the onset of cooling. In particular, the amplitude was 0.09 ± 0.02 at phase 2, which was significantly smaller than that at baseline (*p* = 0.03). The amplitude at phase 3 was 0.25 ± 0.03, which was significantly higher than the amplitudes at phases 1 and 2 (*p* < 0.001). At 25 °C air temperature (Fig. 5b), the wavelet amplitude of endothelial NO-independent activity was 0.14 ± 0.02 at baseline and tended to increase from the onset of cooling. The amplitude at phase 2 was 0.47 ± 0.05, which was significantly higher than that at baseline (*p* < 0.001). Thereafter, even though the amplitude at phase 3 was significantly higher than that at baseline (*p* < 0.001), the amplitude tended to decrease compared with that at phase 2. This tendency was similar to those observed for endothelial NO-independent activity at 20 °C air temperature (Fig. 5a). The amplitude of endothelial NO-dependent activity was 0.35 ± 0.02 at baseline, and there were no significant differences in the amplitudes between baseline and each phase, similar to the results at 20 °C air temperature (Fig. 5a). The amplitude of neurogenic activity at baseline was 0.34 ± 0.02 and tended to decrease from the onset of cooling, similar to the behavior at 20 °C air temperature. The amplitude at phase 2 was significantly smaller than that at baseline (*p* < 0.001) and slightly smaller than that at phase 1. The amplitude at phase 3 was slightly higher than that at phase 2 but was significantly smaller than that at baseline (*p* = 0.02). The amplitude of myogenic activity was 0.17 ± 0.01 at baseline. Similarly, the activity tended to decrease from the onset of cooling, and the amplitude at phase 2 was significantly smaller than that at baseline (*p* < 0.001). Thereafter, the amplitude tended to increase at phase 3 but was still smaller than that at baseline. With regard to the difference in air temperature, particularly at phase 3, the amplitude of NO-independent activity was significantly higher (*p* = 0.007) and the amplitude of myogenic activity was significantly lower (*p* < 0.001) at 25 °C air temperature.

## Discussion

In this study, we focused on the initial stage of CIVD through wavelet analysis of finger skin blood flow signals. Our results indicated that endothelial NO-independent activity was significantly higher and neurogenic and myogenic activities were significantly lower prior to CIVD (phase 2) than at baseline. On the other hand, there were no significant differences in endothelial NO-dependent activity between baseline and all phases at both air temperatures, suggesting that this activity may not be implicated in CIVD. In particular, in this study, healthy participants immersed their fingers in 5 °C water at air temperatures of 20 °C and 25 °C. A previous study reported that the CIVD response was influenced by thermal sensations and that participants wearing T-shirts, shorts, trunks, and socks tended to feel “neither cool nor warm, and comfortable” at an air temperature of 25 °C and “slightly cool and comfortable” at 20 °C [28]. Changes in wavelet amplitudes of the activities showed a similar tendency at both air temperatures, and differences in air temperature were found only at phase 3 (during CIVD), suggesting the same mechanism of CIVD. However, endothelial NO-independent activity was significantly higher and neurogenic activity was significantly lower during vasoconstriction than at baseline at 20 °C air temperature. Additionally, the differences in both activities between vasoconstriction and prior to CIVD were significant. These results indicated that the significant increase of endothelial NO-independent activity and the significant decrease of neurogenic activity at vasoconstriction and prior to CIVD may contribute to the high increment ratio of blood flow at CIVD at 20 °C air temperature.

A previous study [24] reported that the amplitude of neurogenic activity decreased at peak vasoconstriction and decreased further at CIVD. In contrast, endothelial NO independent and NO-dependent activities decreased at peak vasoconstriction and did not change at CIVD [24]. The authors also reported that the fluctuations of wavelet amplitude in neural activity were consistently synchronized in real-time with changes in blood flow, indicating that neural mechanisms and sympathetic withdrawal in particular lead to CIVD. On the other hand, the onset of CIVD is supposed to be related to a reduction in sympathetic drive and norepinephrine release, leading to an opening of the AVAs [14]. Therefore, in this study, we defined three phases from the onset of cooling to the first maximum blood flow prior to CIVD (Fig. 1): vasoconstriction (phase 1), prior to CIVD (phase 2), and CIVD (phase 3). In particular, we speculated that physiological activity at phase 2 contributes considerably to the CIVD event. In the results, we found that the wavelet amplitude of endothelial NO-independent activity at phase 2 was significantly higher (Fig. 5). Similarly, the amplitude of neurogenic activity decreased significantly at phase 2. When phase 2 and phase 3 were not divided, particularly at 20 °C air temperature, the significant differences were not observed due to the low amplitude of endothelial NO-independent activity and high amplitude of neurogenic activity at phase 3. Our analysis showed not only a significant decrease in neurogenic activity but also a significant increase in endothelial NO-independent activity.

Our results showed that neurogenic activity decreased at phase 1 and decreased further at phase 2, which suggested that the decrease in neurogenic activity may contribute to CIVD, consistent with a previous study [24]. Vasoconstriction with skin cooling is dependent on sympathetic noradrenergic nerve function [32]. One possible mechanism for CIVD is the withdrawal of the neurogenic tone innervating the smooth muscle [24]. Indeed, our results showed that not only neurogenic but also myogenic activities decreased at phase 2 at air temperatures of 20 °C and 25 °C compared with baseline. On the other hand, in this study, endothelial NO-independent activity increased at phase 1 and increased further at phase 2. These results suggested that the increase in this activity may also contribute to CIVD, which is not consistent with the previous study [24]. This difference may be due to differences in cooling temperature. The water temperature was 5 °C in this study and 8 °C in the previous study [24]. A higher cooling temperature may induce a moderate CIVD, and the increment of finger temperature at CIVD is larger with a lower cooling temperature [33]. Therefore, in our study, both neurogenic activity and endothelial NO-independent activity may have been involved in CIVD. On the other hand, as there were no significant differences in endothelial NO-dependent activity between baseline and all phases, this activity may not contribute to CIVD, consistent with the previous study [24]. The previous study reported that endothelium-dependent relaxation in the rat mesenteric vascular bed was heterogeneous and that NO was a major component of large arteries (diameter 650 μm), and endothelium-derived hyperpolarization factor (EDHF), which is attributed to endothelial NO-independent activity, may play a role in small arteries (diameter 200 μm) [34]. Even though AVAs are different from mesenteric vascular arteries, the inner diameter of AVAs ranges from 10 to 150 μm [25], and there may be no association between endothelial NO-dependent activity and CIVD.

Our results showed that endothelial NO-independent activity increased significantly at 20 °C air temperature. The activity also increased at 25 °C ambient temperature at phase 1 when blood flow decreased and vasoconstriction occurred due to local cooling. However, a previous study reported that this activity decreased during vasoconstriction [24]. As mentioned above, EDHF is attributed to endothelial NO-independent activity. In rat mesenteric arteries, vascular endothelium acts to depress methoxamine-induced vasoconstriction by releasing EDHF [35]. The cooling temperature (8 °C) was higher in the previous study [24] than that (5 °C) in our study, and CIVD at a higher cooling temperature may be moderate [33], as mentioned earlier. Therefore, the depression of excess vasoconstriction may be unnecessary, and the endothelial NO-independent function may not have been activated in the previous study [24]. Additionally, our results showed that neurogenic activity decreased significantly at 20 °C air temperature and also decreased at 25 °C air temperature at phase 1 of vasoconstriction. This phenomenon seems to be contrary intuitively because finger skin vasoconstriction results from a reflex increase in sympathetic output. However, this result is consistent with that from a previous study [24], and the authors speculated that the expression of α2c-adenoceptor, but not neural activity, may increase [24]. On the other hand, particularly at 20 °C air temperature, endothelial NO-independent activity was lower, and neurogenic and myogenic activities were significantly higher in phase 3 than in phase 2. The underlying mechanism of this behavior is unclear but probably contributes to excess vasodilation and/or vasoconstriction after CIVD. At a minimum, neurogenic activity is superimposed on myogenic activity during the regulation of blood pressure through regulation of the diameter of the vessels [20]. Our results demonstrated that neurogenic and myogenic activities showed a similar trend in both air temperatures, specifically a decrease in phases 1 and 2 compared with baseline and an increase in phase 3 compared with phase 2. On the contrary, the trend of endothelial NO-independent activity seemed to be opposite to that observed from the onset of cooling to CIVD.

The difference in finger temperatures at baseline between air temperatures of 20 °C and 25 °C is high, in spite of the difference in air temperature of 5 °C (Fig. 2a). The AVAs presumably remain closed at ambient water temperatures below 21.5 °C [25], and cooling of the ambient temperature from 25 °C to 17 °C induced a larger drop in skin temperature (average decrease in skin temperature per 1 °C decrease in ambient temperature) than cooling from 32 °C to 25 °C [36]. Although the ratio of the decrease in skin temperature was higher in our study, our result showed that blood flow at baseline was significantly lower at 20 °C air temperature (Fig. 3a), and we speculated that the vasoconstriction/vasodilation of AVAs leads to the huge difference in skin temperature at baseline.

This study had several limitations. We controlled the air temperature at 20 °C and 25 °C by an air conditioner. However, preliminary measurements showed that the measured air temperatures were 21.0 °C ± 0.7 °C and 24.9 °C ± 0.2 °C at 20 °C and 25 °C, respectively. The measured values of air humidity were 36.6 ± 5.5% and 28.8 ± 1.5%, respectively, and this difference was not significant. We controlled the water temperature at 5 °C by a conventional recirculating chiller with a resolution of 0.5 °C. In addition, subjects’ thermal sensation is influenced by clothing insulation as well as air temperature. Therefore, to achieve the similar thermal sensation to the previous study [28], we decided the clothing insulation (e.g., T-shirt and shorts) and air temperatures of 20 °C and 25 °C. On the other hand, compared with other studies at similar conditions, participants may wear lightly and the clothing insulation may be lower.

## Conclusions

In this study, we focused on the initial stage of CIVD of finger skin blood flow at air temperatures of 20 °C and 25 °C through wavelet analysis. The changes in wavelet amplitude showed a similar tendency at both air temperatures. However, when the finger skin temperature was much lower and AVAs might be closed at baseline at 20 °C air temperature, endothelial NO-independent activity was significantly higher, and neurogenic activity was significantly lower at vasoconstriction than at baseline. Additionally, the differences in both activities between vasoconstriction and prior to CIVD were significant, suggesting that the increase of endothelial NO-independent activity and the decrease of neurogenic activity may contribute to the high increment ratio of blood flow at CIVD at 20 °C air temperature. On the other hand, there were no significant differences in endothelial NO-dependent activity between baseline and all phases at either air temperature, suggesting that this activity might not be involved in CIVD.

## Data Availability

The datasets used and/or analyzed during the current study are available from the corresponding author on reasonable request.

## Abbreviations

AVAs: Arteriovenous anastomoses
CIVD: Cold-induced vasodilation
CIVD onset_sbf: The onset of the CIVD event based on skin blood flow
CIVD onset_sk: The onset of the CIVD event based on skin temperature
EDHF: Endothelium-derived hyperpolarization factor
EDV: Endothelial-dependent vasodilation
LDF: Laser Doppler flowmetry
NO: Nitric oxide
*δ*_*max*_: Maximum gradient between minimum and maximum blood flow
*δ*_*ave*_: Averaged gradient between minimum and maximum blood flow
